# Effective Extraction of Limonene and Hibaene from Hinoki (*Chamaecyparis obtusa*) Using Ionic Liquid and Deep Eutectic Solvent

**DOI:** 10.3390/molecules26144271

**Published:** 2021-07-14

**Authors:** Rina Yasutomi, Riki Anzawa, Masamitsu Urakawa, Toyonobu Usuki

**Affiliations:** 1 Department of Materials and Life Sciences, Faculty of Science and Technology, Sophia University, 7-1 Kioicho, Chiyoda-ku, Tokyo 102-8554, Japan; seijikurin52@gmail.com (R.Y.); r.anzawa.4g3@gmail.com (R.A.); 2 Ebisu Kagaku Co. Ltd., 1-10-6 Kajicho, Chiyoda-ku, Tokyo 101-0044, Japan; urakawa@ebisukagaku.com

**Keywords:** *Chamaecyparis obtusa*, extraction, hibaene, ionic liquid, deep eutectic solvent

## Abstract

The essential oils of hinoki (*Chamaecyparis obtusa*) leaves have anti-bacterial, anti-fungal, and relaxation properties that are likely associated with the major components such as sabinene, α-terpinyl acetate, limonene, elemol, myrcene, and hibaene. The present study describes the use of a cellulose-dissolving ionic liquid (IL) [C_2_mim][(MeO)(H)PO_2_] and low-toxicity solvents called betaine-based deep eutectic solvents (DESs) for the efficient extraction of hinoki essential oils. As a control method, organic solvent extraction was performed using either hexane, ethyl acetate (EtOAc), or acetone at 30 °C for 1 h. Both the experimental and control methods were conducted under the same conditions, which relied on partial dissolution of the leaves using the IL and DESs before partitioning the hinoki oils into the organic solvent for analysis. Quantitative analysis was performed using gas chromatography–mass spectrometry (GC-MS) in selected ion monitoring (SIM) mode. The results indicated that extraction using the [C_2_mim][(MeO)(H)PO_2_]/acetone bilayer system improved the yields of limonene and hibaene, 1.5- and 1.9-fold, respectively, when compared with the control method. In addition, extraction using betaine/l-lactic acid (molar ratio 1:1) gave the greatest yields for both limonene and hibaene, 1.3-fold and 1.5-fold greater, respectively, than when using an organic solvent. These results demonstrate the effective extraction of essential oils from plant leaves under conditions milder than those needed for the conventional method. The less toxic and environmentally begin DESs for the extraction are also applicable to the food and cosmetic industries.

## 1. Introduction

**Figure 1:**
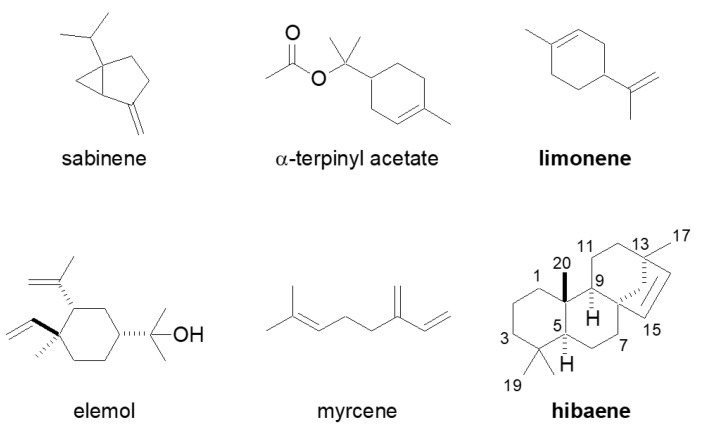
Major components in hinoki leaf oil.

Hinoki (*Chamaecyparis obtusa*) is a conifer of the Cupressaceae family distributed in Japan, China, and other Asian countries. The amount of essential oils in leaves is less than that in the wood. However, the leaf oils possess activities such as anti-bacterial, anti-fungal, and relaxation properties [1,2]. Therefore, the leaf oil has been used to treat urinary tract infections and asthma [1,3]. The major components of the essential oil in hinoki leaves are sabinene, α-terpinyl acetate, limonene, elemol, myrcene, and hibaene (Figure 1) [1].

Ionic liquids (ILs) are organic salts comprised solely of ions and are liquid at temperatures below 100 °C [4]. Due to their low volatility, low flammability, and reusability, ILs are considered environmentally friendly (“green”) solvents. The anion and cation pairs can also be designed to adjust the polarity, viscosity, and electrochemical conductivity of the ILs, and even to reduce their toxicity. The ionic nature of ILs allows them to form hydrogen bonds with biopolymers such as cellulose, lignin, and chitin. As a result, ILs can dissolve these biopolymers, which are generally considered waste products [5,6]. The dissolution of these materials enables more time-efficient and milder methods of refinement into valuable and sustainable materials. For comparison, the conventional method for dissolving cellulose is the viscose process, which requires large amounts of carbon disulfide, sodium hydroxide, sulfuric acid, and fresh water. In addition, this process generates large quantities of wastewater and alkali and acidic pollution [6,7].

The IL 1-ethyl-3-methylimidazolium methylphosphonate [C_2_mim][(MeO)(H)PO_2_] possesses excellent cellulose-dissolving properties under mild conditions [8], and increases the essential oil extraction yield from leaves [9] and wood [10]. However, ILs have varying toxicity and can be difficult to scale up for industrial processes. In addition, ILs can also make purification difficult and can be expensive [11].

Fortunately, new solvents analogous to ILs, called deep eutectic solvents (DESs), have the interesting features of ILs along with unique advantages, as reported by Abbott and co-workers in 2003 [12]. The DESs can be obtained by mixing a hydrogen-bond donor and hydrogen-bond acceptor that form an eutectic mixture with a melting point lower than either of the individual components. Unlike ILs, which are held together by ionic bonding, DESs are held together by hydrogen bonding. The DESs have physical properties similar to those of ILs, but they are relatively nontoxic and inexpensive [13,14].

Betaine, or trimethylglycine, is an amino acid derivative that occurs naturally in plants. Betaine also has anti-inflammatory, anti-ageing, and moisturizing properties [15,16]. Betaine-based DESs have been developed to effectively extract catechins and flavanols from green tea, and preserve the extract over a longer period of time when compared to aqueous and organic solvent extractions [17]. The extraction of flavonoids from Scutellariae Radix using betaine-based DESs was also reported recently [18]. Furthermore, the components of the DES itself may act as active ingredients in cosmetic applications. In the present study, to develop a more eco-friendly method for leaves, essential oil extraction [C_2_mim][(MeO)(H)PO_2_] and betaine-based DESs were used to extract hinoki oils.

## 2. Results and Discussion

### 2.1. Extraction Yields Using IL

The yields from IL-assisted extractions are compared with control in Table 1. A schematic depiction of the results is shown in Figure S1. Using the current IL method, IL/acetone gave the greatest yields for both limonene and hibaene, 1.5-fold and 1.9-fold greater, respectively, than when using organic solvent extraction.

The greatest yields of limonene occurred when IL/acetone and acetone alone were used for extraction, perhaps due to better acetone solubility compared to that in the other organic solvents. For hibaene, control experiments suggested that EtOAc would be the better solvent and gave a better extraction yield. However, when IL was used, the average yield of hibaene was slightly greater in IL/acetone than in IL/EtOAc, perhaps because the IL mixed more easily with acetone than with ethyl acetate, enabling a better stirring efficiency.

### 2.2. Extraction Yields Using DESs

Yields from extractions using DESs are compared with the control in Table 2. A schematic depiction of the results is shown in Figure S2. Using the current DES method, BetLac&H_2_O/acetone gave the greatest yields of both limonene and hibaene, 1.3-fold and 1.5-fold greater, respectively, than when using organic solvent extraction.

The greatest yields of limonene occurred when BetLac&H_2_O/acetone and acetone alone were used for extraction, perhaps due to better solubility in acetone than in other organic solvents. For hibaene, control experiments suggested that EtOAc would be a better solvent than acetone and, thus, give a better extraction yield. However, when DESs were used, the yield of hibaene, on average, was slightly greater in BetLac&H_2_O/acetone than in BetLac&H_2_O/EtOAc. In addition, when the other DESs were used, the extraction yields of both limonene and hibaene depended on the DES used, likely due to the influence of the polarity and viscosity of the DES and the organic solvent, and affinity with the extracted compounds.

## 3. Materials and Methods

### 3.1. General

**Figure 2:**
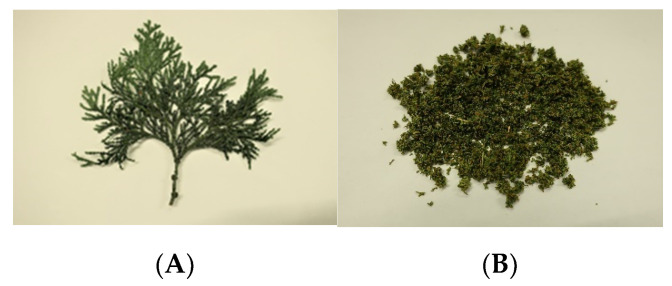
(**A**) Hinoki leaf; (**B**) Hinoki leaf powder.

All reagents were obtained from commercial suppliers and were used without further purification unless otherwise mentioned. The [C_2_mim][(MeO)(H)PO_2_] was purchased from Kanto Chemical (Tokyo, Japan). (*R*)-(+)-Limonene was obtained from Fujifilm Wako Pure Chemical (Tokyo, Japan). Hinoki leaves were purchased from Konishi Noen (Tokushima, Japan), and were powdered with a mixer and then stored at −24 °C prior to use (Figure 2). Gas chromatography–mass spectrometry (GC-MS) analysis was conducted using a Shimadzu GCMS-QP2010 SE instrument with an Agilent DC-WAX capillary column (30 m length, 0.25 mm internal diameter, 0.25 μm film thickness). Gel permeation chromatography (GPC) purification was conducted using an LC-9201 instrument (Japan Analytical Industry, Tokyo, Japan) with JAIGEL-1HR and JAIGEL-2HR columns eluted with chloroform.

Optical rotations were measured on a JASCO P-2200 digital polarimeter at the sodium lamp (λ = 589 nm) D line and are reported as follows: [α]_D_^T^ (*c* g/100 mL, solvent). ^1^H and ^13^C nuclear magnetic resonance (NMR) spectra were recorded on a JEOL JNM-ECA 500 spectrometer (500 MHz). ^1^H NMR data are reported as follows: chemical shift (*δ*, ppm), integration, multiplicity (s, singlet; d, doublet; t, triplet; q, quartet; m, multiplet), coupling constants (*J*) in Hz, assignments. ^13^C NMR data are reported in terms of chemical shift (*δ*, ppm). Fast atom bombardment (FAB)-MS spectra with a magnetic sector for high-resolution measurements were observed on the JEOL JMS-700 and are reported in mass-to-charge ratio (*m*/*z*).

### 3.2. Isolation of Hibaene

To prepare a calibration curve of hibaene, extraction and isolation of hibaene were conducted. The hinoki leaves (*Chamaecyparis obtusa*) (700 g) were extracted for three days at room temperature using EtOAc (3100 mL). After filtration, the EtOAc extract was evaporated and fractioned by column chromatography on silica gel and eluted with 400 mL of hexane, 600 mL of hexane/EtOAc (9:1), and 510 mL of hexane/EtOAc (8:2), yielding three fractions. Fraction 1 (5.9463 g) was analyzed by GC-MS, which confirmed the existence of hibaene.

Furthermore, fraction 1 (5.9463 g) was separated using normal-phase medium-pressure liquid chromatography (NP-MPLC) by eluting with 700 mL of hexane, to yield 35 fractions. Fractions 1–10 (0.16381 g) were analyzed by GC-MS to confirm hibaene, and purification by GPC was conducted to obtain 52 mg of hibaene with 97% purity (Figure S3). The compound was identified as hibaene by high-resolution mass spectrometry (HRMS), and 1D and 2D NMR (^1^H, ^13^C, DEPT, ^1^H-^1^H COSY, HMQC, HMBC, Figures S4–S11).

Hibaene: [α]_D_^25^ −37.6 (*c* 0.1, CHCl_3_); ^1^H NMR (500 MHz, CDCl_3_) *δ* 5.70 (1H, d, *J =* 5.0 Hz, H15), 5.45 (1H, d, *J =* 5.0 Hz, H14), 1.64–1.43 (6H, m, H3/1/7/6/11/16), 1.38–1.22 (7H, m, H6/7/12/3/11/12/2/2), 1.16–1.10 (1H, dt, *J* = 13.0, 5.0 Hz, H12), 0.99 (5H, m, H16/17/9), 0.86–0.80 (8H, m, H19/5/1/18), 0.74 (3H, s, H20); ^13^C NMR (100 MHz, CDCl_3_) *δ* 136.4 (C14), 135.5 (C15), 61.4 (C16), 56.2 (C5), 53.0 (C9), 49.2 (C8), 43.8 (C13), 42.3 (C12), 39.4 (C1), 37.5 (C3), 33.9 (C19), 33.4 (C2/6), 25.1 (C17), 22.1 (C18), 20.3 (C7/11), 18.8 (C12), 15.2 (C20); FAB-HRMS (*m*/*z*) calcd for C_20_H_32_ [M]^+^ 272.2504, found 272.2475.

### 3.3. Organic Solvent Extraction

Organic solvent extraction was performed as a control (Scheme S1) using 40 mg of hinoki leaf powder that was placed into a 1.5 mL Eppendorf tube. Then, 422 µL of organic solvent was added and extraction was conducted at 30 °C and 1500 rpm for 1 h. Extraction was continued for another 5 min to match conditions used for IL-assisted extraction. After removing the stirring chip, the mixture was centrifuged for 1 min and the supernatant collected for analysis. Each extraction was performed three times.

### 3.4. IL-Assisted Extraction

As shown in Scheme S2, extraction using [C_2_mim][(MeO)(H)PO_2_] IL was performed as follows: 0.04 g of hinoki leaf powder was placed into a 1.5 mL Eppendorf tube. Then, 211 μL of IL and 211 µL of organic solvent were added, and extraction was conducted at 30 °C and 1500 rpm for 1 h. After this, 211 μL of H_2_O was added and diluted with 633 μL of EtOAc and stirred for 5 min at 1500 rpm. This mixture then was centrifuged for 1 min. After collecting the organic layer, the IL/leaf layer was washed twice with 633 μL of EtOAc. The combined organic layers were used for GC-MS analysis. Each extraction was performed three times.

### 3.5. Preparation of Deep Eutectic Solvents (DESs)

Three HBDs (glycerol, l-lactic acid, and sucrose) were used in combination with HBA (betaine) to produce the DESs (Table 3).

Betaine and glycerol were added to a round-bottom flask. The mixture was stirred at 80 °C for 1 d. The resulting material was a white liquid, which was diluted 2:1 with water to reduce viscosity before extraction; this caused the solution to become colorless.

Betaine and l-lactic acid were added to a round-bottom flask. BetLac was obtained by heating with stirring at 80 °C for 1 d with the addition of a small amount of water, followed by evaporation at 50 °C using a rotatory evaporator. The resulting solution was diluted 2:1 with water to reduce viscosity before extraction.

Betaine and sucrose were added to a round-bottom flask. BetSuc was obtained by heating with stirring at 50 °C for 1 d with the addition of a small amount of water, followed by evaporation at 50 °C using a rotatory evaporator. The resulting solution was diluted 2:1 with water to reduce viscosity before extraction.

### 3.6. Extraction Using DESs

As shown in Scheme S3, extraction using DESs was performed under the same conditions as IL-assisted extraction.

### 3.7. GC-MS Quantification

The GC-MS analysis was performed as follows: the oven temperature was held at 45 °C for 2 min, then raised at a rate of 30 °C/min until it reached 220 °C, and it was held at this temperature for another 30 min. The conditions were as follows: carrier gas: helium; flow rate: 1.7 mL/min; pressure: 98.1 kPa; injector temperature: 250 °C; splitting ratio: 1:26; interface temperature: 250 °C; EI-MS source temperature: 200 °C; solvent: hexane, EtOAc or acetone; injection volume: 5.0 μL.

Calibration curves were obtained as follows: Standard solutions of (*R*)-(+)-limonene and isolated hibaene were prepared. A β-ionone internal standard (IS) was added to each solution of IS (*X*_i_), (*R*)-(+)-limonene (*X*_l_), and hibaene (*X*_h_). Analysis of the injected solution provided peak areas for IS (*Y*_i_), (*R*)-(+)-limonene (*Y*_l_), and hibaene (*Y*_h_), which were used to determine the ratio of analyte and IS concentration *x* (*x* = *X*_l or h_/*X*_i_), and the ratio of analyte and IS peak areas *y* (*y* = *Y*_l or h_/*Y*_i_). The calibration equations are:

limonene:

*y* = 0.1042*x* − 0.0005 (*R*^2^ = 0.9988) (1)

hibaene:

*y* = 0.2474*x* + 0.0005 (*R*^2^ = 0.9995) (2)

Quantitative analysis was conducted in selected ion monitoring (SIM) mode. A fixed concentration of the IS was added to the analyte. Injection of this solution gave peak areas *Y*_i,_ *Y*_l_, and *Y*_h_, to allow calculation of *y*. Using this value in the calibration equations gave *x*, from which the concentrations of (*R*)-(+)-limonene (*X*_l_) and hibaene (*X*_h_) could be determined. The amount of analyte (mg) in the extract solution could be determined using 3 mL of total extract solution with an injection amount of 5.0 μL. The wt% extraction yield was then calculated as the percentage of analyte in the extract (mg) over the original amount of leaf powder (mg).

## 4. Conclusions

Hinoki leaves contain essential oils with anti-bacterial, anti-fungal, and relaxation properties. The isolation of hibaene from hinoki (*Chamaecyparis obtusa*) leaves and essential oil extraction using the [C_2_mim][(MeO)(H)PO_2_] IL and betaine-based DESs were accomplished. Limonene and hibaene were chosen as the target compounds for quantitative analysis.

Hibaene (52 mg) was isolated from hinoki leaves (700 g) by the use of CC, NP-MPLC, and GPC. Organic solvent extractions using hexane, EtOAc, and acetone were used as controls. The results demonstrated that acetone extraction produced the greatest limonene yield, while EtOAc was best for limonene extraction among the control methods. The IL-assisted extraction method relies first on partial dissolution of the hinoki leaves in the IL, followed by partitioning of the oil into an organic solvent, and finally separation by centrifugation. Results revealed that IL/acetone gave the best yields of limonene and hibaene, with 1.5- and 1.9-fold greater yields, respectively, than those using organic solvent extraction controls.

In addition, extractions by DESs were performed under the same conditions as those for IL-assisted extraction, which revealed that BetLac&H_2_O/acetone gave the greatest yields for both limonene and hibaene, 1.3-fold and 1.5-fold greater, respectively, than when using organic solvent extraction. The less toxic and environmentally begin DESs for the extraction would be much applicable to the food and cosmetic industries.

A further study for the extraction of essential oils from plant materials utilizing betaine-based DESs is currently underway in our laboratory.

## Figures and Tables

**Table 1:** Extraction yields for limonene and hibaene (*n* = 3).

Extraction Solvent	Average Yield (%, *w*/*w*)	
	Limonene	Hibaene
hexane	0.02923 ± 0.01067	0.00423 ±0.00035
EtOAc	0.08763 ± 0.00884	0.00667 ± 0.00047
acetone	0.09758 ± 0.00475	0.00577 ± 0.00032
IL IL/hexane	0.06511 ±0.01695 0.09903 ± 0.02795	0.00732 ± 0.000205 0.00671 ± 0.00097
IL/EtOAc	0.12501 ± 0.03357	0.00954 ± 0.00156
IL/acetone	0.14782 ± 0.01978	0.01068 ± 0.00036

**Table 2:** Extraction yields for limonene and hibaene (*n* = 3).

Extraction Solvent	Average Yield (%, *w*/*w*)	
	Limonene	Hibaene
hexane	0.02923 ± 0.01067	0.00423 ± 0.00035
EtOAc	0.08763 ± 0.00884	0.00667 ± 0.00047
acetone	0.09758 ± 0.00475	0.00577 ± 0.00032
BetGly&H_2_O/hexane BetGly&H_2_O/EtOAc	0.05112 ± 0.00032 0.07961 ± 0.00754	0.00481 ± 0.00026 0.00695 ± 0.00047
BetGly&H_2_O/acetone	0.07434 ± 0.00401	0.00568 ± 0.00030
BetLac&H_2_O/hexane	0.06574 ± 0.00474	0.00668 ± 0.00030
BetLac&H_2_O/EtOAc	0.11201 ± 0.00572	0.00903 ± 0.00125
BetLac&H_2_O/acetone	0.12451 ± 0.00702	0.00934 ± 0.00060
BetSuc&H_2_O/hexane	0.07493 ± 0.00138	0.00845 ± 0.00072
BetSuc&H_2_O/EtOAc	0.10342 ± 0.00260	0.00882 ± 0.00089
BetSuc&H_2_O/acetone	0.06147 ± 0.00537	0.00502 ± 0.00038

**Table 3:** DESs prepared for extraction.

DES	HBA	HBD	Molar Ratio
BetGly	betaine (Bet)	glycerol (Gly)	1:1
BetLac	betaine (Bet)	l-lactic acid (Lac)	1:1
BetSuc	betaine (Bet)	sucrose (Suc)	2:1

Betaine:glycerol = 1:1 DES [19]; Betaine:l-lactic acid = 1:1 DES [20]; Betaine:sucrose = 2:1 DES [21].

## Data Availability

Not applicable.

## Supplementary Materials

The following are available online, Experimental schemes (Schemes S1–S3); Bar graph of extraction results (Figures S1 and S2); GC-MS chromatograph (Figure S3); NMR spectra (Figures S4–S11).

## Sample Availability

Samples of the isolated compounds hibaene is available from the authors.
