# Supplementary material for: Effective Extraction of Limonene and Hibaene from Hinoki (Chamaecyparis obtusa) Using Ionic Liquid and Deep Eutectic Solvent
Source: Molecules. 2021 Jul 14;26(14):4271. doi: 10.3390/molecules26144271 (PMC8303359; doi:10.3390/molecules26144271)
Supplement: Supplementary file 1 [file molecules-26-04271-s001.zip › molecules-1279404-supplementary.pdf]

Supplementary data

Effective Extraction of Limonene and Hibaene from Hinoki (*Chamaecyparis obtusa*) using Ionic Liquid and Deep Eutectic Solvent

Rina Yasutomi,[1] Riki Anzawa,[1] Masamitsu Urakawa,[2] and Toyonobu Usuki[1,]*

[1] *Department of Materials and Life Sciences, Faculty of Science and Technology, Sophia University, 7-1 Kioicho, Chiyoda-ku, Tokyo 102-8554, Japan*

[2] *Ebisu Kagaku Co. Ltd, 1-10-6 Kajicho, Chiyoda-ku, Tokyo 101-0044, Japan*

Corresponding author: t-usuki@sophia.ac.jp

Contents

S2-S3: Schemes of extraction

S4: Bar graph of extraction results using IL and DESs

S5: GC-MS chromatogram

S6-S9: NMR spectrum of hibaene

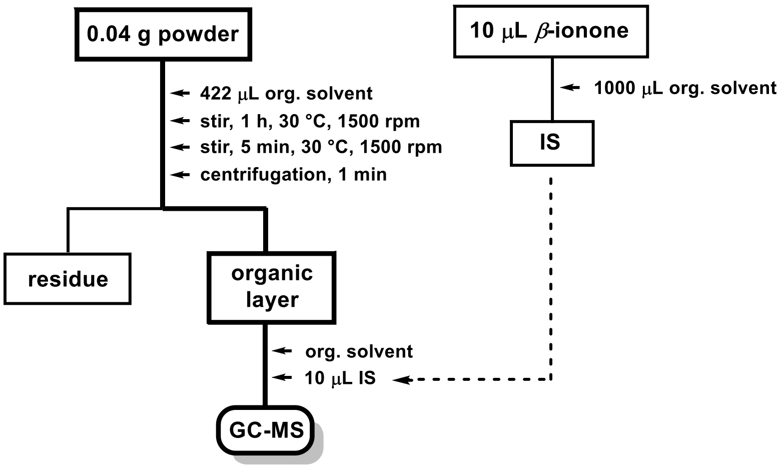

Scheme S1. Extraction using organic solvent as a control.

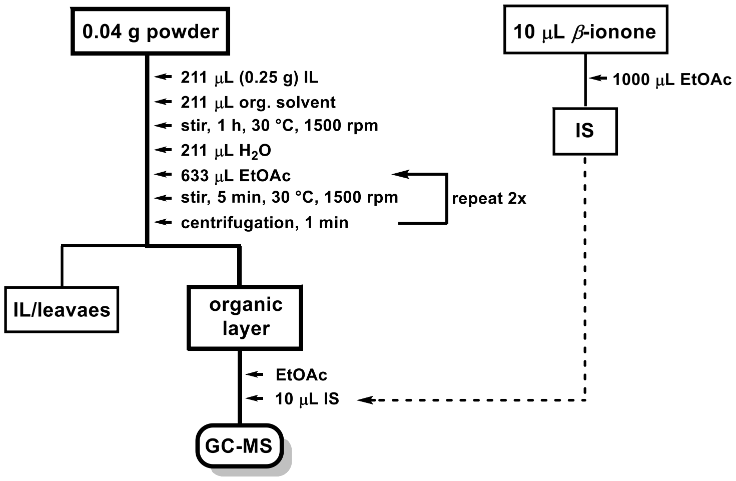

Scheme S2. IL-assisted extraction procedure.

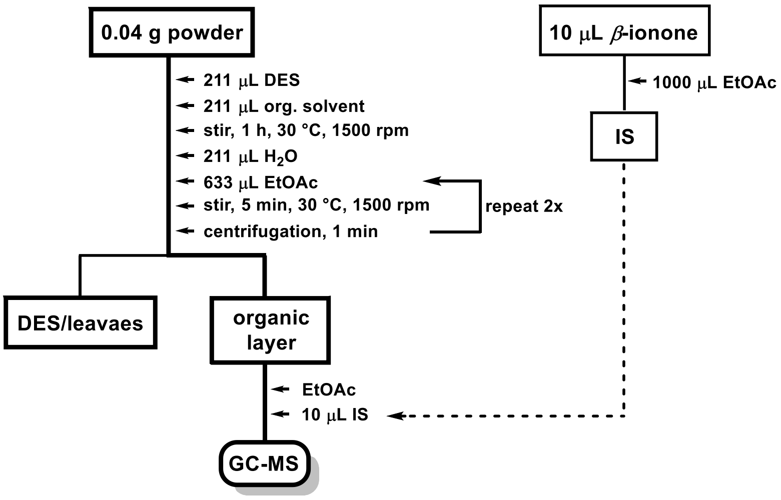

Scheme S3. Extraction using DESs.

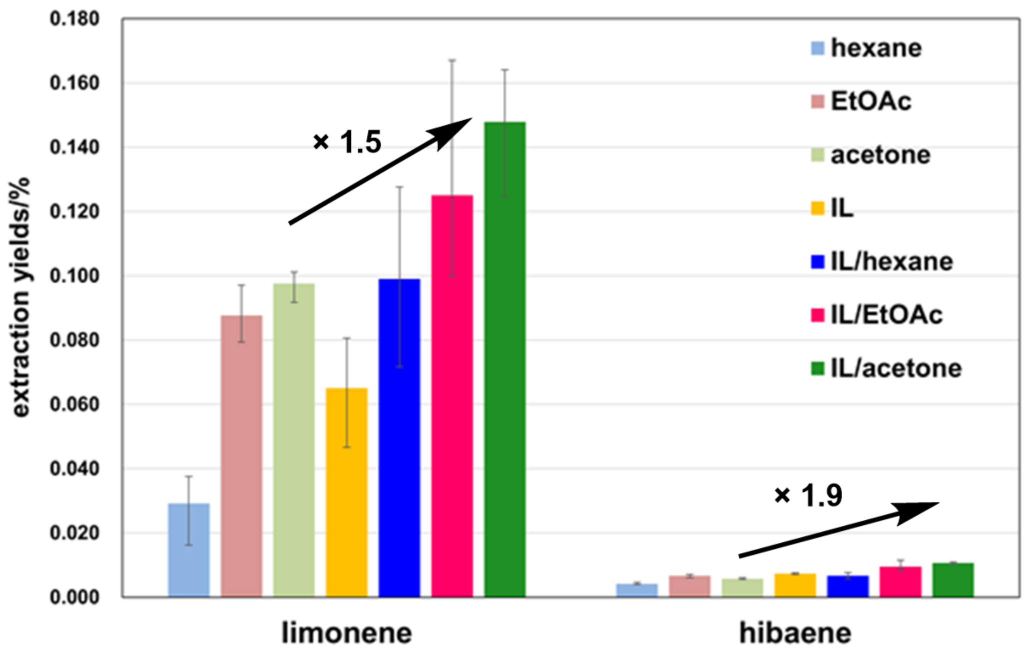

Figure S1. Bar graph of extraction results using IL.

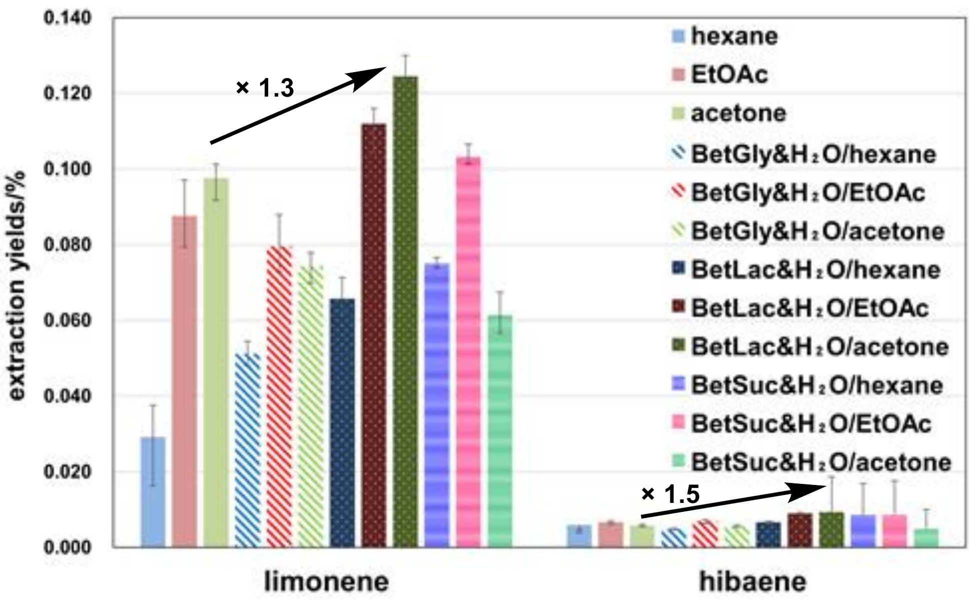

Figure S2. Bar graph of extraction results using DESs.

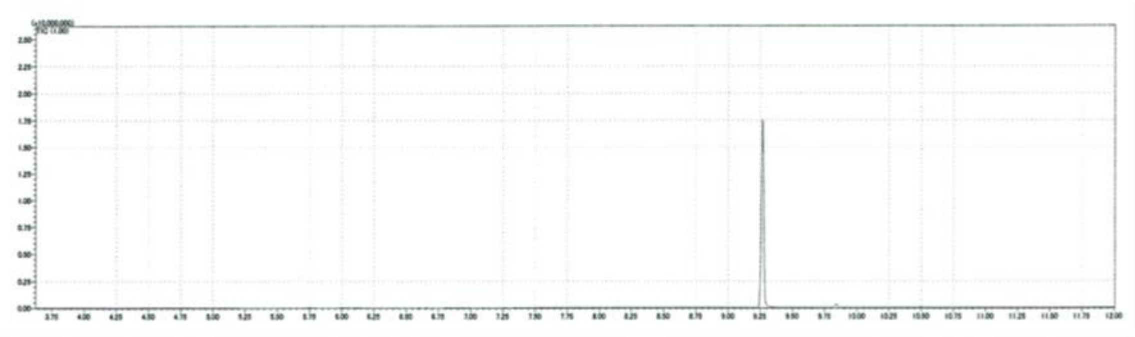

Figure S3. Chromatogram of Fr.1-10 of hibaene (9.27 min).

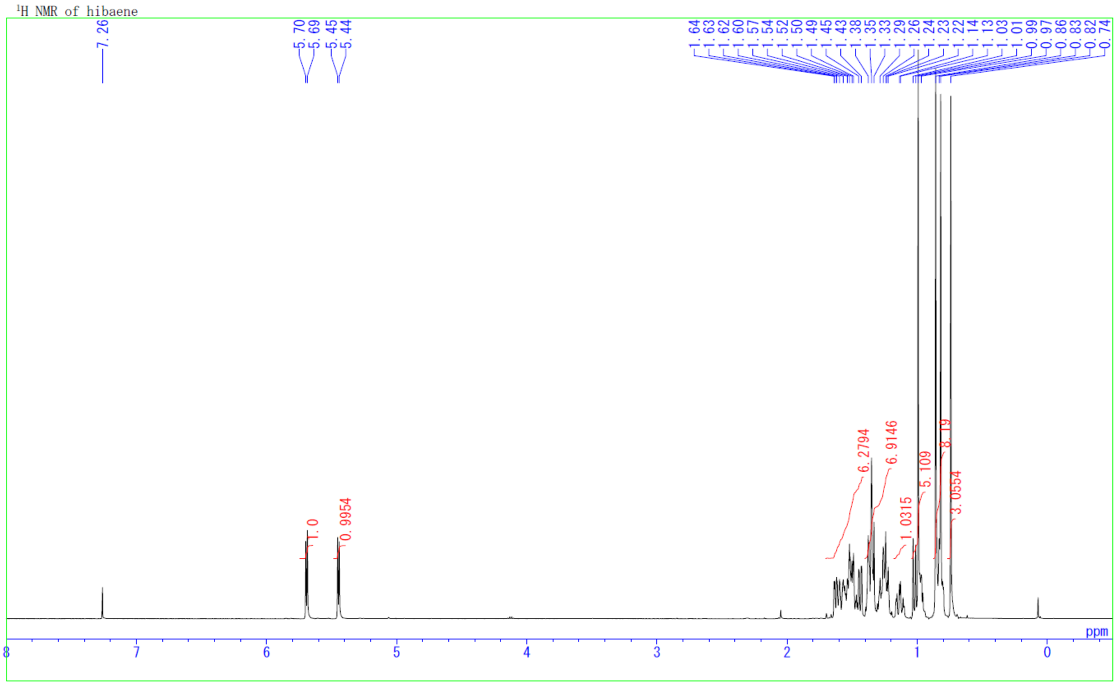

Figure S4. ^{1}H NMR spectrum of hibaene.

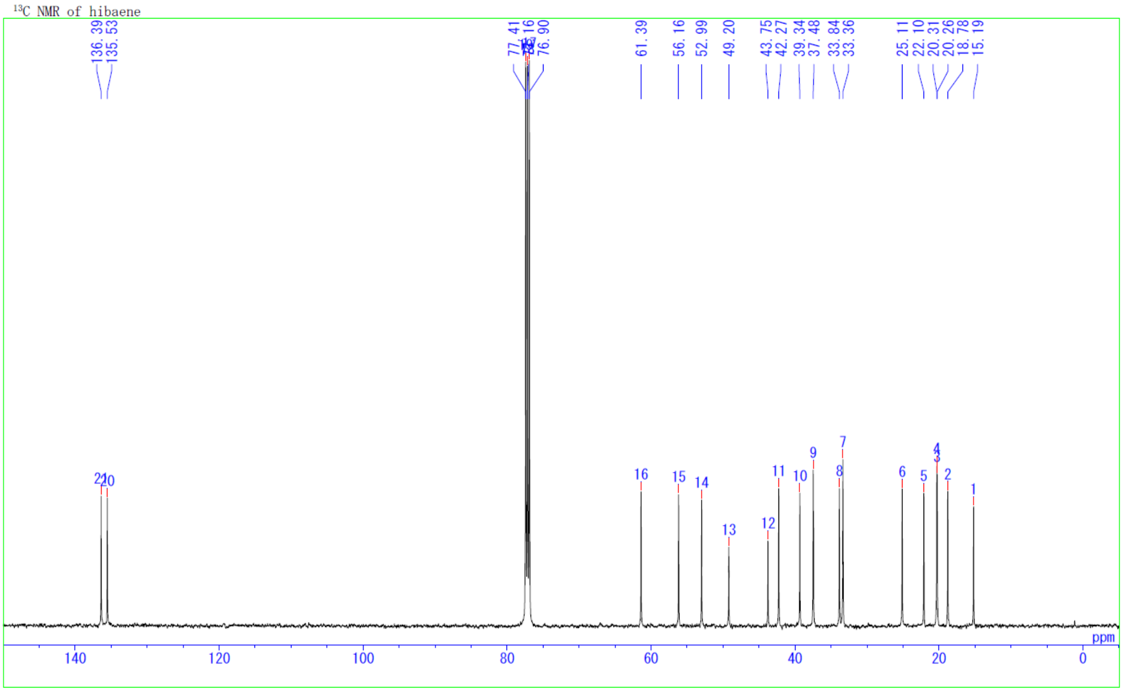

Figure S5. ^{13}C NMR spectrum of hibaene.

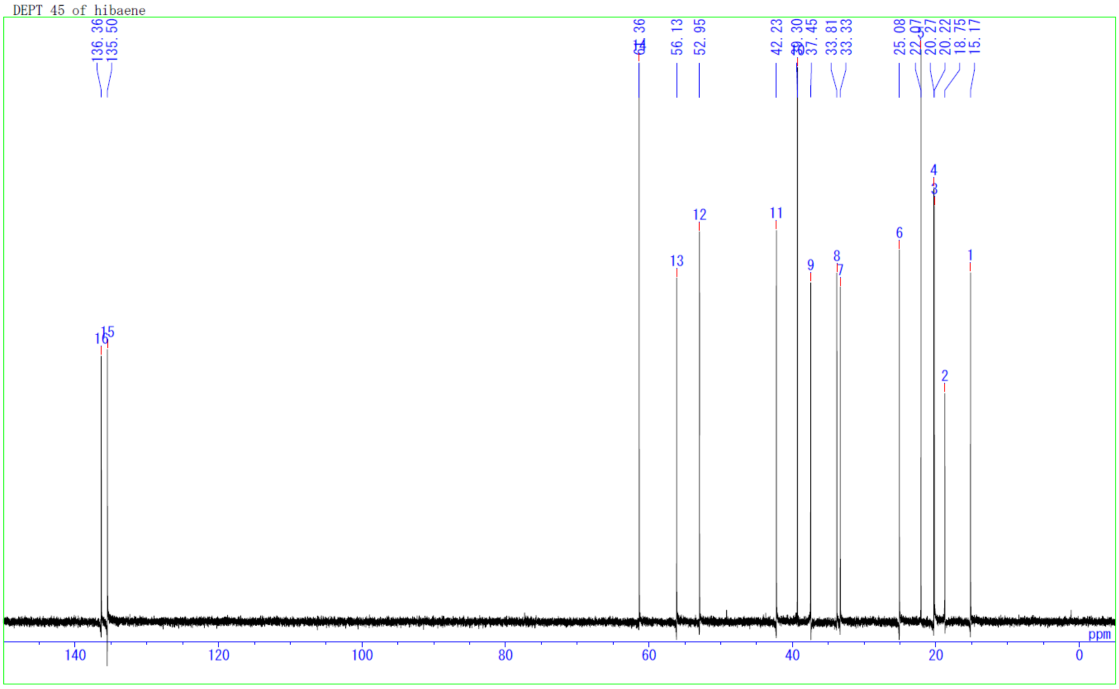

Figure S6. DEPT 45° spectrum of hibaene.

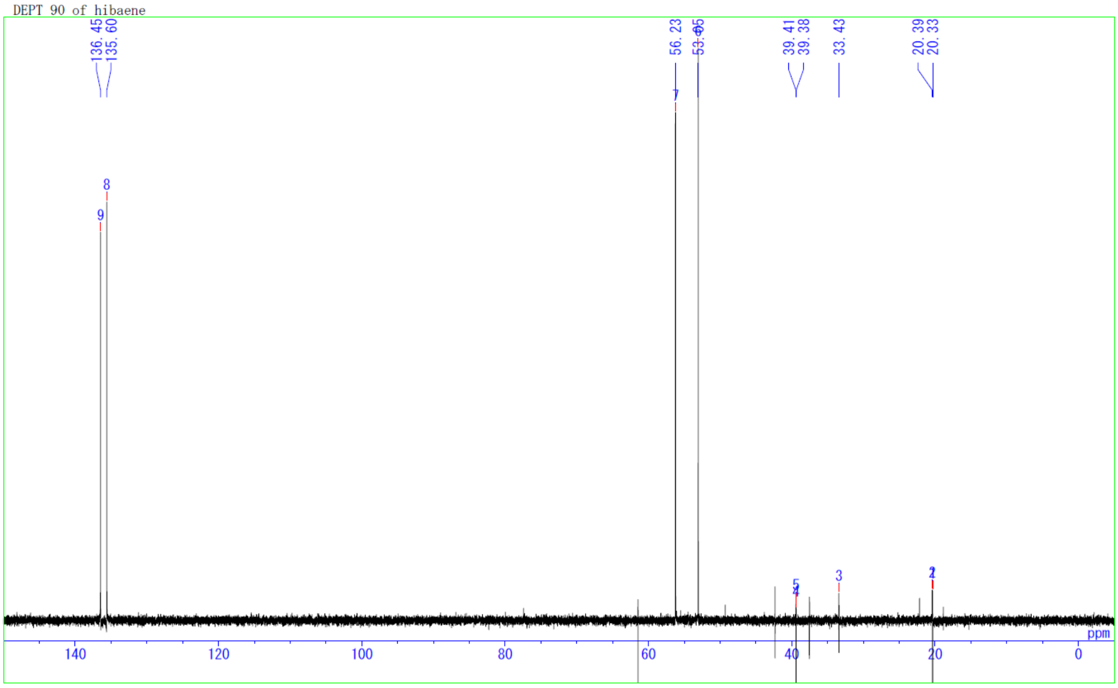

Figure S7. DEPT 90° spectrum of hibaene.

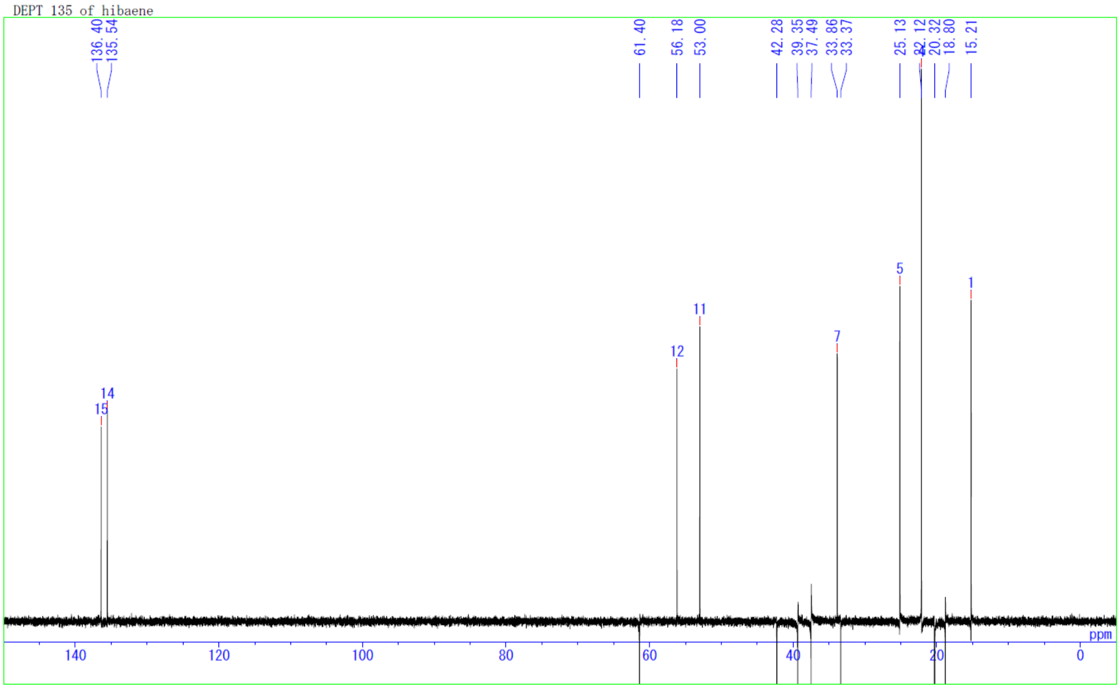

Figure S8. DEPT 135° spectrum of hibaene.

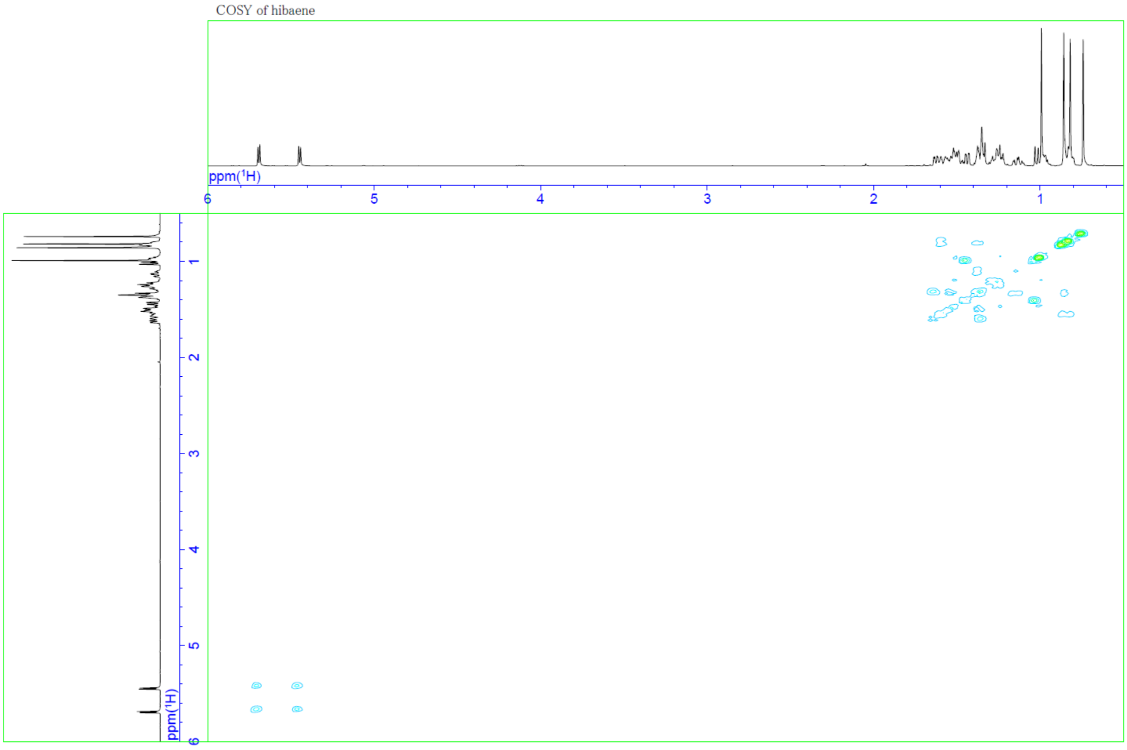

Figure S9. ^{1}H-^{1}H COSY of hibaene.

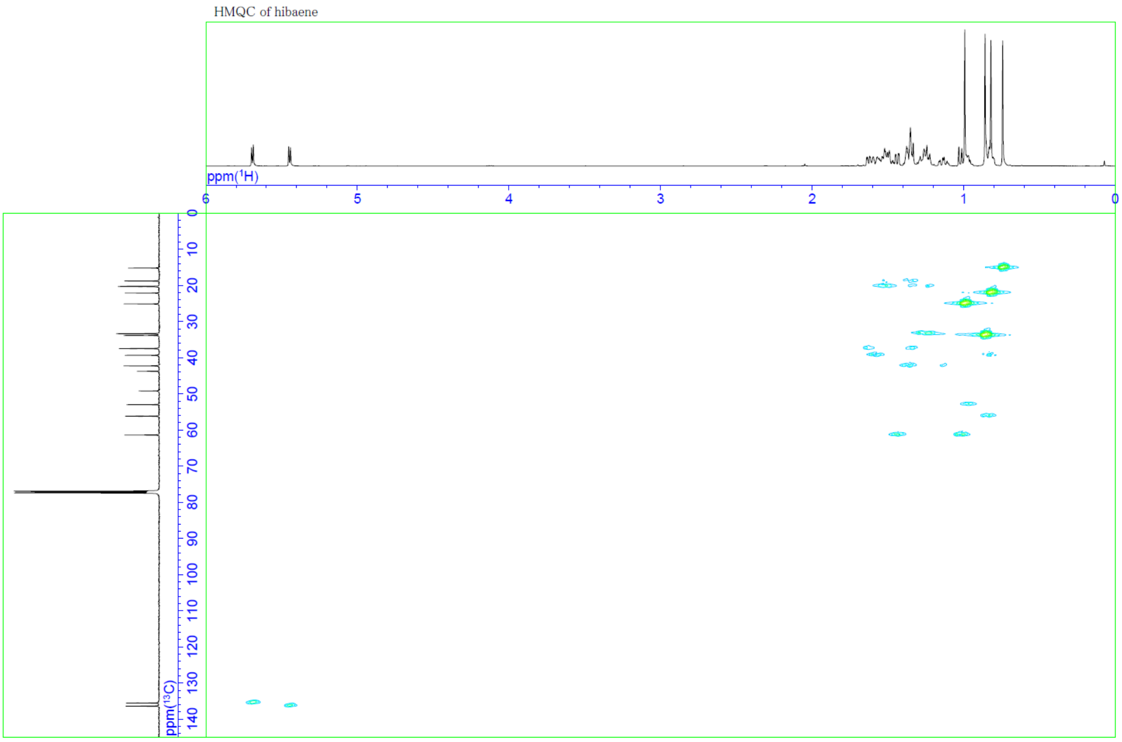

Figure S10. HMQC of hibaene.

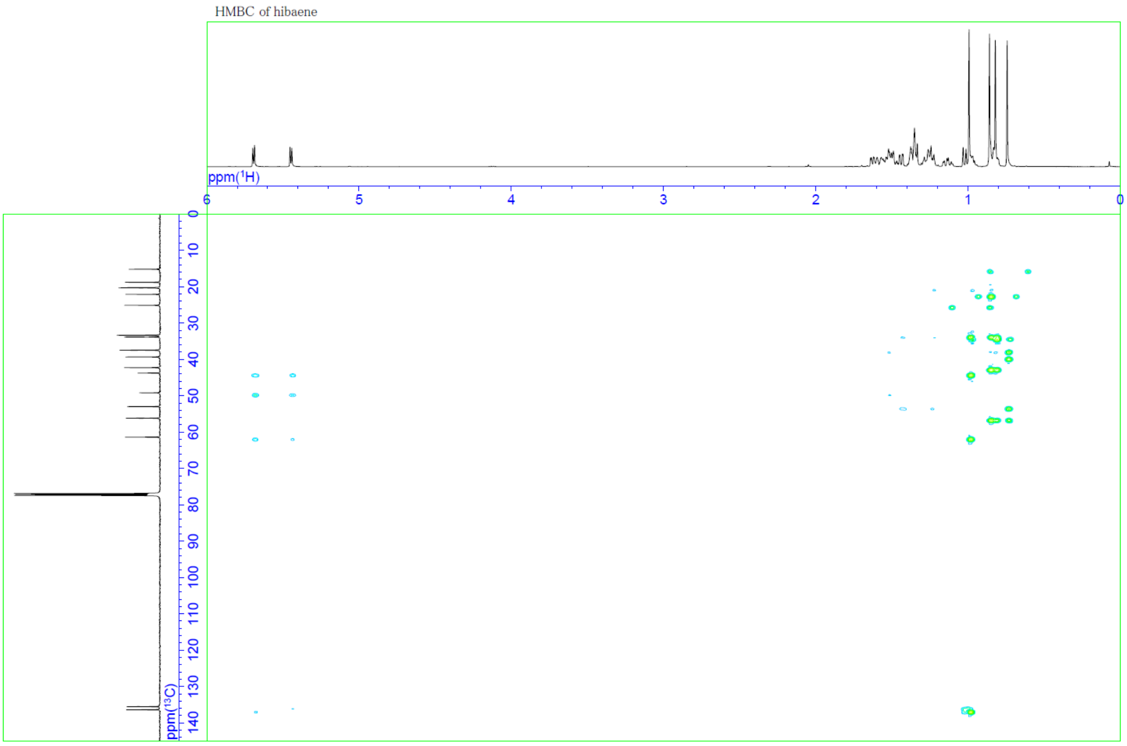

Figure S11. HMBC of hibaene.
